# Genetic Diversity of Arginine Catabolic Mobile Element in *Staphylococcus epidermidis*

**DOI:** 10.1371/journal.pone.0007722

**Published:** 2009-11-06

**Authors:** Maria Miragaia, Herminia de Lencastre, Francoise Perdreau-Remington, Henry F. Chambers, Julie Higashi, Paul M. Sullam, Jessica Lin, Kester I. Wong, Katherine A. King, Michael Otto, George F. Sensabaugh, Binh An Diep

**Affiliations:** 1 Laboratory of Molecular Genetics, Instituto de Tecnologia Química e Biológica, Universidade Nova de Lisboa (ITQB/UNL), Oeiras, Portugal; 2 Laboratory of Microbiology, The Rockefeller University, New York, New York, United States of America; 3 Division of Infectious Diseases, Department of Medicine, University of California San Francisco, San Francisco, California, United States of America; 4 Veterans Affairs Medical Center and University of California San Francisco, San Francisco, California, United States of America; 5 Division of Infectious Diseases, School of Public Health, University of California, Berkeley, California, United States of America; 6 Laboratory of Human Bacterial Pathogenesis, National Institute of Allergy and Infectious Diseases, National Institute of Health, Bethesda, Maryland, United States of America; National Institute of Allergy and Infectious Diseases, National Institutes of Health, United States of America

## Abstract

**Background:**

The methicillin-resistant *Staphylococcus aureus* clone USA300 contains a novel mobile genetic element, arginine catabolic mobile element (ACME), that contributes to its enhanced capacity to grow and survive within the host. Although ACME appears to have been transferred into USA300 from *S. epidermidis*, the genetic diversity of ACME in the latter species remains poorly characterized.

**Methodology/Principal Findings:**

To assess the prevalence and genetic diversity of ACME, 127 geographically diverse *S. epidermidis* isolates representing 86 different multilocus sequence types (STs) were characterized. ACME was found in 51% (65/127) of *S. epidermidis* isolates. The vast majority (57/65) of ACME-containing isolates belonged to the predominant *S. epidermidis* clonal complex CC2. ACME was often found in association with different allotypes of staphylococcal chromosome cassette *mec* (SCC*mec*) which also encodes the recombinase function that facilities mobilization ACME from the *S. epidermidis* chromosome. Restriction fragment length polymorphism, PCR scanning and DNA sequencing allowed for identification of 39 distinct ACME genetic variants that differ from one another in gene content, thereby revealing a hitherto uncharacterized genetic diversity within ACME. All but one ACME variants were represented by a single *S. epidermidis* isolate; the singular variant, termed ACME-I.02, was found in 27 isolates, all of which belonged to the CC2 lineage. An evolutionary model constructed based on the eBURST algorithm revealed that ACME-I.02 was acquired at least on 15 different occasions by strains belonging to the CC2 lineage.

**Conclusions/Significance:**

ACME-I.02 in diverse *S. epidermidis* isolates were nearly identical in sequence to the prototypical ACME found in USA300 MRSA clone, providing further evidence for the interspecies transfer of ACME from *S. epidermidis* into USA300.

## Introduction

*Staphylococcus epidermidis* is a ubiquitous commensal of the human skin and mucosal surfaces and a major cause of indwelling medical device infections. This organism is notorious for its capacity to accumulate antibiotic resistance determinants and to produce biofilm, making infections caused by this opportunistic pathogen particularly difficult to treat. The large gene pool of antibiotic resistance and virulence determinants in *S. epidermidis* is shared with other more pathogenic species such as *S. aureus*. In particular, multidrug-resistant conjugative plasmids and staphylococcal chromosome cassette *mec* (SCC*mec*) elements conferring β-lactam resistance are transferred frequently between *S. epidermidis* and *S. aureus*, enabling rapid evolution and adaptation against antibiotic selection pressure [1], [2], [3].

Many *S. epidermidis* strains also carry the arginine catabolic mobile element (ACME), a novel genomic island that may contribute to enhanced capacity of this species to colonize the human skin and mucosal surfaces [4]. The horizontal transfer of ACME from *S. epidermidis* to *S. aureus* is thought to be central in the evolution of the highly transmissible community-associated methicillin resistant *S. aureus* (CA-MRSA) clone USA300 [4]. In USA300, ACME is integrated at the *orfX* site downstream of SCC*mec*, and is flanked by repeat sequences typical of SCC cassettes [4], [5]. Mobilization of ACME is believed to be mediated by the cassette chromosome recombinases (*ccrAB*) encoded by SCC element [5], [6]. This element has been found also in diverse *S. aureus* genetic backgrounds, suggesting frequent horizontal dissemination [4], [5], [6], [7]. ACME contains two gene clusters (*arc* encoding a secondary arginine deiminase system and *opp-3* encoding an ABC transporter) that are homologs of virulence determinants in other bacterial species [5]. However, ACME was shown not to contribute to the severity of necrotizing pneumonia or skin abscess using rat infection models [8], a finding consistent with the presence of this element among various commensal *Staphylococcus* species [4], [9]. Using highly sensitive *in vivo* competition assays, ACME was shown to confer bacterial survival advantage in a rabbit bacteremia model and a mouse gastrointestinal colonization model [5], [10]. Taken together, the data suggest that ACME may play a role in bacterial transmission among susceptible hosts by contributing to bacterial growth and survival.

Although ACME was found in diverse *S. epidermidis* [4], the genetic diversity of ACME among *S. epidermidis* has not yet been characterized. It is also not clear the extent to which *S. epidermidis* serves as a reservoir of ACME for horizontal transfer to related pathogenic species such as *S. aureus*. We report herein an analysis of 127 genotypically diverse isolates of *S. epidermidis* from worldwide sources. The results show extensive genetic variations within ACME. ACME allotypes are often found in association with diverse allotypes of SCC*mec*. The most prevalent allotype of ACME among *S. epidermidis* is nearly identical to prototypical ACME found in USA300, suggesting that ACME transfers from *S. epidermidis* into the epidemic USA300 clone.

## Materials and Methods

### Bacterial Isolates

A representative collection of *S. epidermidis* strains was selected to include isolates as diverse as possible, in terms of genetic background, geographic source and temporal origins. The study collection comprised 127 *S. epidermidis* strains, 93 methicillin-resistant *S. epidermidis* (MRSE) and 34 methicillin-susceptible *S. epidermidis* (MSSE), isolated between 1996 and 2005 in 18 different countries: Argentina (3), Bulgaria (3), Cape Verde (8), China (1), Colombia (2), Denmark (29), Greece (4), Hungary (2), Iceland (20), Italy (2), Japan (1), Mexico (5), Poland (2), Portugal (8), Spain (1), Taiwan (2), Uruguay (1) and USA (33). The collection selected included 79 isolates from human carriage, 40 from disease and 8 isolates for which no information on the clinical origin was available.

### Restriction Fragment Length Polymorphism (RFLP) Analysis of Arginine Deiminase (*arc*) Gene Cluster

Chromosomal DNA of *S. epidermidis* strains was digested with *Cla*I and the resulting fragments were separated by a electrophoresis in a 1% agarose gel in 1x Tris-acetate-EDTA buffer at 30 volts for 17 h. DNA fragments were transferred by vacuum blotting to nitrocellulose membranes as previously described [11] and hybridized with DNA probes for a region encompassing ACME-encoded *arcC* and *arcB* genes using primers arcC-1, 5′-AATTTATCAGCCTGCTCTTTTGT-3′, and arcB-1, 5′-AAAACAGGTAATCCACATACA-3′. High stringency hybridization was performed with ECL direct prime labelling and detection systems (Amersham Biosciences, Buckinghamshire, U.K.), using a wash buffer with 0.1 X Standard Sodium Citrate (SSC). The different ClaI-*arcCB* hybridization band patterns found in this study were identified using arabic numbers (e.g., ClaI-*arcCB* types 1–7).

### ACME PCR Scan

*S. epidermidis* isolates were screened for the presence of ACME using PCR-based assays using the primer pairs AIPS.27-AIPS.28 (*arcA*) and AIPS.45-AIPS.46 (*opp3A*), as previously described [5]. The *arcA* and *opp-3A* genes are surrogate markers of the *arc* gene cluster encoding for an arginine deiminase system and *opp-3* encoding for an ABC transporter system, respectively. *S. epidermidis* isolates containing *arcA* and/or *opp-3* clusters were further characterized by a PCR-based scanning/tiling method that allows for identification of variations in ACME gene content and gene synteny, as previously described [5]. This method is based on 31 individual PCR reactions using distinct primer pairs designed to generate 1–2 kb PCR fragments that overlap with one another at both ends for complete scanning coverage of the prototype ACME found in USA300. PCR scan patterns were classified into three ACME allotypes: (1) ACME-I contains both the *arc* and *opp-3* gene clusters; (2) ACME-II contains *arc* but not *opp-3*; and (3) ACME-III contains *opp-3* but not *arc*. Distinct PCR scan patterns within each ACME allotype are given subtype designations (e.g. ACME-I.02). Sequencing of the PCR amplicons from the PCR scan among 5 ACME-I.02-positive *S. epidermidis* isolates from different countries were performed using primers as previously described [5].

### SCC*mec* Typing

*S. epidermidis* isolates were characterized for the two central elements of the staphylococcal cassette chromosome *mec* (SCC*mec*), namely, the *ccr* complex encoding for recombinases and the *mec* complex encoding for broad spectrum β-lactam resistance. The multiplex PCR strategy, M-PCR 1, was used to identify the 5 types of *ccr* gene complex, and M-PCR 2 to identify class A to class C *mec* complex, as previously described [12].

### *Ccr*-mediated Excision of ACME

A tetracycline-selectable temperature-sensitive plasmid, pSR2, containing the *ccrAB2* gene complex, was electroporated into *S. epidermidis* strain 1457. Se1457(pSR2) was passaged for three days in tryptic soy broth (TSB) supplemented with 10 µg/ml of tetracycline at 30°C. Growth at the non-permissive temperature of 42°C in antibiotic-free TSB resulted in loss of pSR2 in the excision mutants. Individual colonies were screened for excision and loss of ACME by assaying for the loss of the *arcA* gene. Confirmation of ACME excision was performed by Southern hybridization of *Sma*I DNA restriction fragments after pulsed-field gel electrophoresis with the probe encompassing *arcC* and *arcB* as described above [13]. *In vitro* growth rate of *S. epidermidis* 1457 and its isogenic ACME-excision mutant was determined in tryptic soy broth as measured by OD_600_.

### MLST and eBURST

Multilocus sequence typing (MLST) was performed, based on the sequencing of internal fragments of seven housekeeping genes, and using the revised scheme described by Thomas et *al.* [14]. The most likely patterns of evolutionary descent in the collection were assessed using the eBURST algorithm (http://eburst.mlst.net), using previously validated parameters [15]. Clonal complexes were represented by the abbreviation CC, and singletons were represented by the abbreviation S. CC2 was subdivided into clusters I and II, and cluster II was further separated into subclusters as previously described [15], [16].

### Construction of Evolutionary Models and Estimation of Independent ACME Acquisitions

An evolutionary model illustrating the number of ACME acquisitions was constructed, based on the evolutionary relationships as defined by eBURST and ACME typing as defined by the PCR scanning strategy (see above). The number of independent ACME acquisitions was estimated based on the following assumptions: (i) there is a low probability of ACME excision, (ii) there is a low probability that the exact same mutation occurs twice; and (iii) for ACME acquisition/excision to occur, a gene coding for a recombinase (*ccrAB* or *ccrC*) must be present in the chromosome. The number of acquisition of SCC elements (SCC*mec* and SCC non-*mec*) was estimated using the same methods.

### Statistical Analysis

Two-sided chi-square test statistics were used for between group comparisons (Stata, version 9, College Station, Texas).

## Results

### Distribution of ACME and SCC*mec* among *S. epidermidis* Lineages

**Figure 1:**
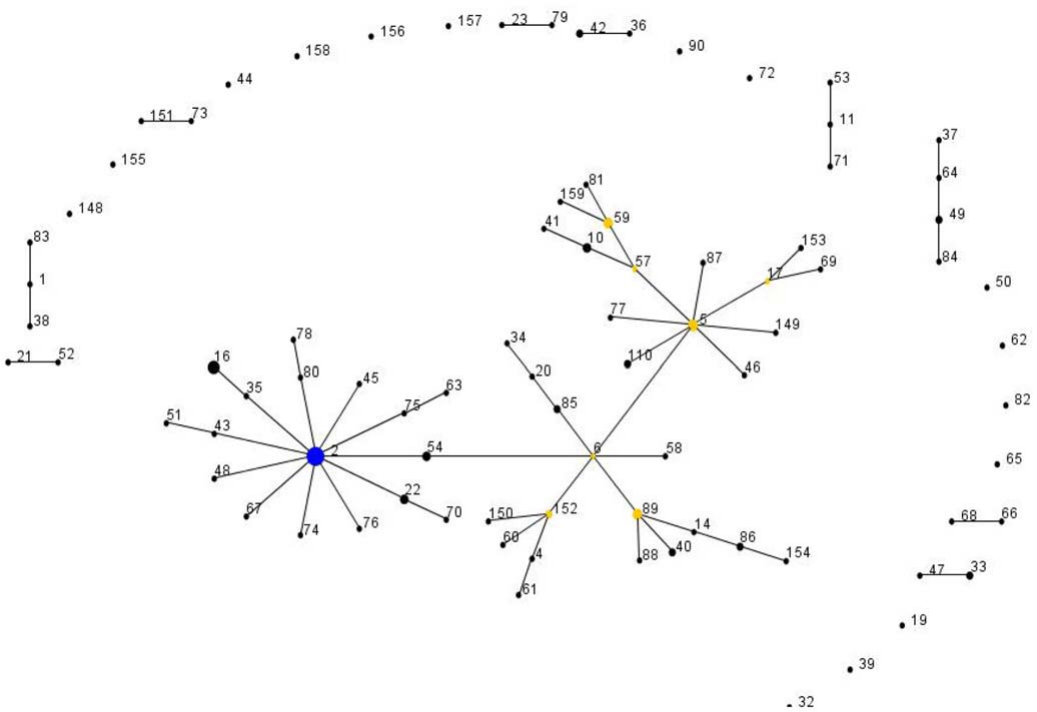
Application of eBURST algorithm to MLST data for the collection of 127 *S. epidermidis* isolates. Each ST is represented by a filled circle. Blue and yellow circles represent STs that are group and sub-group founders, respectively. CC comprised the groups of connected STs, considering that STs have at least 6 alleles in common with at least another ST inside a CC.

Of the 127 *S. epidermidis* isolates selected to represent the broad genetic and geographic diversity of the species, 52% (65/127) contained either the ACME-encoded *arcA* and/or *opp-3* gene clusters. Presence of *arcA* and/or *opp-3* gene clusters did not correlate with isolates recovered from infection sites or colonization sites. Using a revised MLST scheme [14], [16], 86 distinct sequence types (ST) were identified among the 127 isolates (Figure 1 and Table 1). The majority of the STs (50 of 86) were closely related and clustered into a single clonal complex, CC2. Of note, 65% (57/88) of isolates belonging to CC2 contained ACME-encoded *arcA* or *opp-3*, whereas only 21% (8/39) of isolates belonging to non-CC2 clonal complexes contained these genes (*P*<0.001).

**Table 1:** Diversity of ACME and SCC*mec* among *S. epidermidis*.

Strain	Country	Methicillin resistance^1^	CC^2^	MLST^3^	ACME PCR Scan^4^	ClaI-arcC/B pattern^5^	SCCmec typing^6^
DEN112	Denmark	+	2-I	ST2 (7-1-2-2-4-1-1)	ACME-I.02	4	III
ICE091	Iceland	+	2-I	ST2 (7-1-2-2-4-1-1)	ACME-I.02	4	III
DEN049	Denmark	+	2-I	ST2 (7-1-2-2-4-1-1)	ACME-neg	NH	III
DEN102	Denmark	+	2-I	ST2 (7-1-2-2-4-1-1)	ACME-neg	NH	III
DEN121	Denmark	+	2-I	ST2 (7-1-2-2-4-1-1)	ACME-neg	NH	III
ICE027	Iceland	+	2-I	ST2 (7-1-2-2-4-1-1)	ACME-neg	NH	III
DEN167	Denmark	+	2-I	ST2 (7-1-2-2-4-1-1)	ACME-neg	NH	IV
ICE146	Iceland	+	2-I	ST2 (7-1-2-2-4-1-1)	ACME-neg	NH	IV
ICE181	Iceland	+	2-I	ST2 (7-1-2-2-4-1-1)	ACME-neg	NH	IV
DEN071	Denmark	+	2-I	ST2 (7-1-2-2-4-1-1)	ACME-I.02	4	A/ccrAB3,ccrAB4,ccrC
ICE050	Iceland	+	2-I	ST2 (7-1-2-2-4-1-1)	ACME-neg	NH	A/ccrAB3,ccrAB4,ccrC
ICE124	Iceland	+	2-I	ST2 (7-1-2-2-4-1-1)	ACME-neg	NH	A/ccrAB3,ccrAB4,ccrC
BD0917	USA	+	2-I	ST2 (7-1-2-2-4-1-1)	ACME-neg	.	NT/ccrAB3,ccrC
BD0909	USA	−	2-I	ST2 (7-1-2-2-4-1-1)	ACME-neg	.	mecA-neg/ccrAB2
BD0942	USA	+	2-I	ST16 (2-1-2-2-15-1-1)	ACME-I.02	.	C/ccrAB1,ccrAB2,ccrC
BD0944	USA	+	2-I	ST16 (2-1-2-2-15-1-1)	ACME-I.02	.	NT/ccrAB2
BD0907	USA	−	2-I	ST16 (2-1-2-2-15-1-1)	ACME-I.02	.	mecA-neg/ccr-neg
BD0926	USA	−	2-I	ST16 (2-1-2-2-15-1-1)	ACME-I.02	.	mecA-neg/ccr-neg
BD0969	USA	−	2-I	ST16 (2-1-2-2-15-1-1)	ACME-I.02	.	mecA-neg/ccr-neg
BD0905	USA	+	2-I	ST16 (2-1-2-2-15-1-1)	ACME-neg	.	NT/ccrAB2,ccrC
DEN061	Denmark	+	2-I	ST22 (7-1-2-2-4-7-1)	ACME-I.02	8	III
ICE019	Iceland	+	2-I	ST22 (7-1-2-2-4-7-1)	ACME-I.02	4	A/ccrAB2,ccrAB4,ccrC
ICE037	Iceland	+	2-I	ST22 (7-1-2-2-4-7-1)	ACME-I.02	4	A/ccrAB4,ccrC
HFA6014	Portugal	+	2-I	ST35 (2-1-2-2-4-1-1)	ACME-I.02	4	A/ccrC
DEN004	Denmark	+	2-I	ST45 (20-1-2-2-4-1-1)	ACME-I.02	4	IV
DEN087	Denmark	+	2-I	ST48 (7-1-2-2-4-1-4)	ACME-I.02	4	C/ccrAB2,ccrAB4
BD0972	USA	−	2-I	ST54 (1-1-2-2-4-1-1)	ACME-I.02	.	mecA-neg/ccr-neg
DEN109	Denmark	+	2-I	ST54 (1-1-2-2-4-1-1)	ACME-I.02	3	III
AGT18	Argentina	+	2-I	ST63 (22-1-2-2-4-13-1)	ACME-I.02	4	C/ccrAB2
DEN055	Denmark	+	2-I	ST70 (7-1-2-2-14-7-1)	ACME-I.02	4	A/ccrAB4
PLN131	Poland	+	2-I	ST75 (7-1-2-2-4-13-1)	ACME-I.02	4	III
AGT17	Argentina	+	2-I	ST78 (21-1-2-2-4-13-1)	ACME-I.02	4	III
ICE076	Iceland	+	2-I	ST80 (21-1-2-2-4-1-1)	ACME-I.02	4	III
DEN139	Denmark	+	2-I	ST54 (1-1-2-2-4-1-1)	ACME-I.17	6	IV
ICE175	Iceland	+	2-I	ST43 (7-1-2-2-1-1-1)	ACME-neg	NH	A/ccrAB3,ccrAB4,ccrC
COB20	Colombia	+	2-I	ST51 (7-1-2-2-1-1-8)	ACME-neg	NH	IV
DEN036	Denmark	+	2-I	ST67 (7-1-18-2-4-1-1)	ACME-neg	NH	III
ESP43	Spain	+	2-I	ST74 (7-1-2-12-4-1-1)	ACME-neg	NH	A/ccrAB3,ccrAB4,ccrC
GRE28	Greece	+	2-I	ST76 (7-1-2-14-4-1-1)	ACME-neg	NH	A/ccrAB1,ccrAB2,ccrAB3
BD0904	USA	+	2-II	ST5 (1-1-1-2-2-1-1)	ACME-I.02	.	A/ccrC
ICE192	Iceland	+	2-II	ST5 (1-1-1-2-2-1-1)	ACME-I.07	1	IV
DEN002	Denmark	+	2-II	ST5 (1-1-1-2-2-1-1)	ACME-II.07	6	C/ccrAB2,ccrC
BD0902	USA	+	2-II	ST5 (1-1-1-2-2-1-1)	ACME-I.13	.	NT/ccrAB2
BD0922	USA	−	2-II	ST5 (1-1-1-2-2-1-1)	ACME-neg	.	mecA-neg/ccr-neg
ICE009	Iceland	+	2-II	ST6 (1-1-2-2-2-1-1)	ACME-I.08	0	A/ccrAB2,ccrAB3
DEN076	Denmark	−	2-II	ST14 (1-1-2-1-1-1-1)	ACME-I.02	4	mecA-neg/ccr-neg
HFA6181	Portugal	−	2-II	ST17 (1-1-6-2-2-1-1)	ACME-I.02	4	mecA-neg/ccr-neg
CV27	Cape Verde	+	2-II	ST20 (1-1-2-2-1-1-3)	ACME-II.05	3	B/ccrAB2,ccrC
ICE026	Iceland	+	2-II	ST34 (1-1-2-2-1-13-3)	ACME-I.18	6	IV
ICE087	Iceland	+	2-II	ST40 (1-1-2-1-3-1-1)	ACME-I.02	8	A/ccrAB4,ccrC
DEN046	Denmark	−	2-II	ST40 (1-1-2-1-3-1-1)	ACME-I.04	4	mecA-neg/ccr-neg
MCO150	Mexico	+	2-II	ST46 (1-1-1-2-2-1-7)	ACME-II.11	2′	IV
HFA6162A	Portugal	−	2-II	ST57 (1-1-1-1-2-1-1)	ACME-I.06	3	mecA-neg/ccrAB4
CHI35	China	+	2-II	ST59 (2-1-1-1-2-1-1)	ACME-II.04	3	B/ccrAB2,ccrAB4
TAW060	Taiwan	−	2-II	ST59 (2-1-1-1-2-1-1)	ACME-II.12	2	mecA-neg/ccrAB4
BD0912	USA	−	2-II	ST59 (2-1-1-1-2-1-1)	ACME-neg	.	mecA-neg/ccr-neg
BD0950	USA	+	2-II	ST59 (2-1-1-1-2-1-1)	ACME-neg	.	II
GRE34	Greece	+	2-II	ST69 (1-18-6-2-2-1-1)	ACME-I.10	6	IV
BUG43	Bulgaria	+	2-II	ST77 (23-1-1-2-2-1-1)	ACME-II.08	6	C/ccrAB2,ccrC
DEN077	Denmark	+	2-II	ST81 (2-17-1-1-2-1-1)	ACME-II.09	2	B/ccrAB2,ccrAB4
BD0943	USA	+	2-II	ST85 (1-1-2-2-1-1-1)	ACME-II.19	.	V
TAW113	Taiwan	−	2-II	ST85 (1-1-2-2-1-1-1)	ACME-II.13	3	mecA-neg/ccrAB2
BD0937	USA	+	2-II	ST86 (1-2-2-1-1-1-1)	ACME-I.11	.	II
URU23	Uruguay	+	2-II	ST86 (1-2-2-1-1-1-1)	ACME-I.12	6	NT/ccrAB2,ccrAB4
HUR50	Hungary	−	2-II	ST87 (7-1-1-2-2-1-1)	ACME-I.02	4	mecA-neg/ccr-neg
BD0928	USA	−	2-II	ST110 (1-1-1-6-2-1-1)	ACME-I.14	.	mecA-neg/ccrAB2
BD0929	USA	−	2-II	ST110 (1-1-1-6-2-1-1)	ACME-II.15	.	mecA-neg/ccrAB2
BD0936	USA	+	2-II	ST148 (1-1-1-5-2-1-11)	ACME-I.02	.	B/ccrC
BD0915	USA	+	2-II	ST149 (1-1-1-2-2-1-10)	ACME-I.15	.	NT/ccrAB2
BD0931	USA	−	2-II	ST150 (1-1-2-6-2-5-1)	ACME-II.16	.	mecA-neg/ccrAB2
BD0964	USA	−	2-II	ST152 (1-1-2-6-2-1-1)	ACME-II.20	.	mecA-neg/ccrAB2
BD0935	USA	−	2-II	ST152 (1-1-2-6-2-1-1)	ACME-neg	.	mecA-neg/ccr-neg
BD0965	USA	−	2-II	ST153 (2-1-6-2-2-1-1)	ACME-II.21	.	mecA-neg/ccrAB2
BD0946	USA	−	2-II	ST154 (1-2-1-1-1-1-1)	ACME-III.01	.	mecA-neg/ccr-neg
BD0908	USA	−	2-II	ST157 (1-23-3-6-2-1-1)	ACME-I.09	.	mecA-neg/ccr-neg
BD0934	USA	+	2-II	ST159 (2-1-23-1-2-1-1)	ACME-II.18	.	NT/ccrAB2
DEN101	Denmark	+	2-II	ST89 (1-1-2-1-2-1-1)	ACME-III.02	NH	IV
ICE120	Iceland	+	2-II	ST89 (1-1-2-1-2-1-1)	ACME-III.03	NH	IV
BD0948	USA	+	2-II	ST89 (1-1-2-1-2-1-1)	ACME-neg	.	IV
BD0971	USA	+	2-II	ST89 (1-1-2-1-2-1-1)	ACME-neg	.	IV
DEN022	Denmark	+	2-II	ST4 (1-1-6-6-2-1-1)	ACME-neg	NH	IV
DEN208	Denmark	+	2-II	ST10 (1-1-1-1-3-1-1)	ACME-neg	NH	IV
ICE095	Iceland	+	2-II	ST10 (1-1-1-1-3-1-1)	ACME-neg	NH	IV
DEN132	Denmark	+	2-II	ST10 (1-1-1-1-3-1-1)	ACME-neg	NH	B/ccrAB2,ccrAB4
CV47	Cape Verde	+	2-II	ST41 (1-1-1-1-3-1-11)	ACME-neg	NH	IV
BUG46	Bulgaria	+	2-II	ST58 (1-1-2-2-2-13-1)	ACME-neg	NH	IV
MEX060	Mexico	+	2-II	ST61 (2-1-6-6-2-1-1)	ACME-neg	NH	NT/ccrAB2
HFA6286	Portugal	−	2-II	ST88 (1-1-2-1-2-1-7)	ACME-neg	NH	mecA-neg/ccr-neg
DEN019	Denmark	+	1	ST1 (1-2-2-2-1-1-10)	ACME-neg	NH	IV
ICE024	Iceland	+	1	ST38 (1-2-2-5-1-1-10)	ACME-neg	NH	IV
GRE41	Greece	+	1	ST83 (1-2-1-2-1-1-10)	ACME-neg	NH	IV
DEN062	Denmark	+	11	ST11 (3-1-5-5-3-4-11)	ACME-neg	NH	IV
DEN148	Denmark	+	11	ST50 (3-1-5-5-3-77-4)	ACME-neg	NH	IV
CV59	Cape Verde	+	11	ST53 (3-1-5-5-11-4-11)	ACME-neg	NH	IV
CV11	Cape Verde	+	11	ST62 (3-21-5-5-3-4-4)	ACME-neg	NH	NT/ccrAB2,ccrAB4
MEX037	Mexico	+	11	ST71 (3-1-5-5-3-1-11)	ACME-neg	NH	II
DEN185	Denmark	+	21	ST21 (2-1-1-2-1-1-1)	ACME-I.03	4	IV
ICE102	Iceland	+	21	ST52 (2-2-1-2-1-1-1)	ACME-I.16	4	IV
AGT24	Argentina	+	23	ST23 (7-1-2-1-3-3-1)	ACME-neg	NH	III
CV45	Cape Verde	+	23	ST79 (21-1-2-1-3-3-1)	ACME-neg	NH	IV
COB17	Colombia	−	33	ST33 (12-10-5-5-13-5-21)	ACME-neg	NH	mecA-neg/ccr-neg
JAP263	Japan	+	33	ST33 (12-10-5-5-13-5-21)	ACME-neg	NH	C/NT
HUR51	Hungary	+	33	ST47 (12-1-5-5-13-5-21)	ACME-neg	NH	B/ccrAB2
ICE021	Iceland	+	42	ST36 (11-6-2-1-1-13-1)	ACME-neg	NH	I
DEN116	Denmark	+	42	ST42 (1-6-2-1-1-13-1)	ACME-neg	NH	A/ccrAB1
ICE159	Iceland	+	42	ST42 (1-6-2-1-1-13-1)	ACME-neg	NH	B/ccrAB1,ccrC
HFA6173B	Portugal	+	49	ST37 (18-1-5-5-11-4-20)	ACME-neg	NH	IV
DEN094	Denmark	+	49	ST49 (12-1-5-5-3-4-20)	ACME-neg	NH	IV
MEX035	Mexico	+	49	ST49 (12-1-5-5-3-4-20)	ACME-neg	NH	IV
PLN064	Poland	+	49	ST64 (12-1-5-5-11-4-20)	ACME-neg	NH	NT/ccrAB2
DEN176	Denmark	+	49	ST84 (12-2-5-5-3-4-20)	ACME-neg	NH	IV
ITL034	Italy	+	66	ST66 (12-3-5-5-7-14-11)	ACME-neg	NH	IV
DEN110	Denmark	+	66	ST68 (12-3-5-5-7-1-11)	ACME-neg	NH	IV
BD0932	USA	−	S	ST151 (1-5-2-6-2-1-4)	ACME-II.17	.	mecA-neg/ccr-neg
BD0920	USA	−	S	ST155 (1-1-2-1-4-1-10)	ACME-neg	.	mecA-neg/ccr-neg
BD0933	USA	−	S	ST156 (1-2-6-2-1-1-11)	ACME-neg	.	mecA-neg/ccr-neg
BD0910	USA	−	S	ST158 (1-2-6-2-22-1-1)	ACME-II.14	.	mecA-neg/ccr-neg
BUG37	Bulgaria	−	S	ST19 (8-7-12-4-12-2-2)	ACME-neg	NH	mecA-neg/ccr-neg
ITL299	Italy	−	S	ST32 (1-1-7-1-3-5-14)	ACME-II.10	3	mecA-neg/ccr-neg
GRE53	Greece	+	S	ST39 (22-1-5-5-10-13-12)	ACME-III.04	NH	C/ccrAB2
CV28	Cape Verde	−	S	ST44 (1-6-6-2-1-1-1)	ACME-II.06	3	mecA-neg/ccr-neg
HFA6226	Portugal	−	S	ST60 (1-1-2-6-2-1-16)	ACME-neg	NH	mecA-neg/ccr-neg
CV13	Cape Verde	+	S	ST65 (1-19-17-4-9-10-2)	ACME-neg	NH	NT/ccrAB2,ccrAB4
CV20	Cape Verde	+	S	ST72 (8-2-2-4-9-6-9)	ACME-I.05	3	IV
HFA6391	Portugal	−	S	ST73 (1-5-2-6-2-1-6)	ACME-neg	NH	mecA-neg/ccr-neg
MCO151	Mexico	+	S	ST82 (17-20-5-5-3-4-4)	ACME-neg	NH	IV
HFA6096	Portugal	−	S	ST90 (16-1-2-1-2-12-1)	ACME-neg	NH	mecA-neg/ccrC

1 “+” methicillin-resistant *S. epidermidis*; “−”, methicillin-susceptible *S. epidermidis*; ^2^CC, clonal complex, as previously defined by eBURST analysis [15], [16]; S, singleton; ^3^MLST, multilocus sequence typing [14]; 7-loci allelic profile listed in parenthesis (*arcC-aroE-gtr-mutS-pyrR-tpiA-yqiL*); ^4^ACME PCR scan method [5] defines genetic variants within 3 ACME allotypes: ACME-I contains *arc* and *opp-3* gene clusters; ACME-II contains *arc* but not *opp-3*; and ACME-III contains *opp-3* but not *arc*. ACME-neg is negative for both *arc* and *opp-3*. Amplicons from the ACME PCR scan for 5 underlined ACME-I.02 variants were used for DNA sequencing and construction of 25-kb contigs. ^5^NH, no hybridization with *arcCB* probe; *ClaI*-*arcCB* banding patterns shown in Figure 3; ^6^SCC*mec* typing to identify class A, B, C, and other non-typeable (NT) *mec* gene complex, and 5 types of *ccr* gene complex, ccrAB1, ccrAB2, ccrAB3, ccrAB4, ccrC. SCC*mec* type I contains B/ccrAB1; type II A/ccrAB2; type III A/ccrAB3; type IV B/ccrAB2; and type V C/ccrC.

Among MRSE isolates, 58% (54/93) contained SCC*mec* type I through V, and 42% (39/93) non-typeable SCC*mec* elements (Table 1). MRSE contained 1 to 3 different *ccr* gene complexes that could potentially mobilize SCC*mec* and ACME. There is no association between carriage of ACME and carriage of different SCC*mec* allotypes. Among MSSE isolates, 29% (10/34) contained SCC-like elements containing *ccr* genes but lacking the *mecA* gene. In all, 81% (103/127) of *S. epidermidis* isolates carried *ccr* genes.

### Excision of ACME by CcrAB in *S. epidermidis*

**Figure 2:**
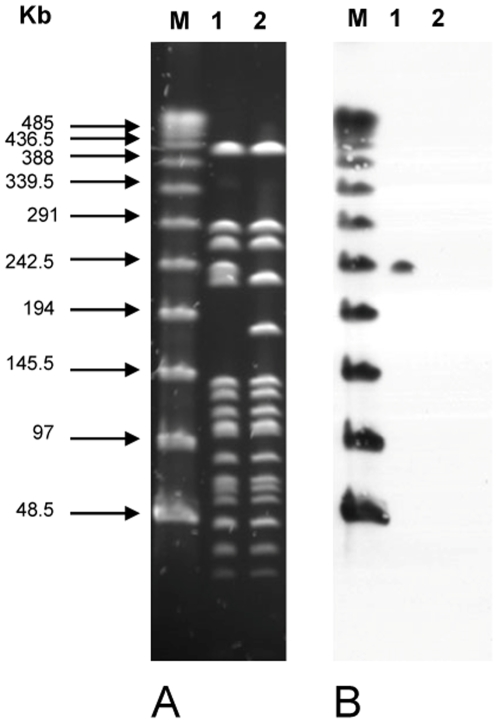
Mobilization of ACME in *S. epidermidis* 1457 by CcrAB. (A) Pulsed-field gel electrophoresis after *Sma*I restriction of chromosomal DNA, for the parental strain *S. epidermidis* 1457 (lane 1) and its ACME excision mutant (lane 2). M = Lambda ladder, and (B) hybridization of *Sma*I restriction patterns of strain 1457 (lane 1) and its ACME excision mutant (lane 2) with a DNA probe for ACME (*arcCB*).

Horizontal transfer of ACME in *S. aureus* is mediated by SCC-encoded cassette recombinases (*ccr*), which catalyze the site-specific recombination between repeat sequences flanking the element and an *attB* site within *orfX* [5]. To test whether SCC-encoded *ccr* could mobilize ACME in *S. epidermidis*, we provided *in trans ccrAB2* via plasmid pSR2 in a clinical isolate *S. epidermidis* 1457 and assayed for excision of ACME by pulsed-field gel electrophoresis of *Sma*I-digested chromosomal DNA and hybridization with ACME-specific *arcCB* probe (Figure 2). A 240-kb *Sma*I fragment hybridized with the arcCB probe in the *S. epidermidis* 1457 parental strain, whereas a corresponding 180-kb fragment did not hybridize with the *arcCB* probe in the ACME-excision mutant. This corresponds to a deletion of approximately 60-kb DNA fragment, which probably contains another SCC-like element mobilizable by CcrAB in addition to ACME (typically 30-kb in size). This is reminiscent of a CcrAB-mediated mobilization of 55-kb of DNA encompassing both SCC*mec* and ACME in *S. aureus* clone USA300 [5]. There was no difference in the *in vitro* growth rate of *S. epidermidis* 1457 and its ACME excision mutant, confirming the finding in *S. aureus* that carriage of ACME does not engender a biological fitness cost [5].

### Genetic Diversity of ACME in *S. epidermidis*

**Figure 3:**
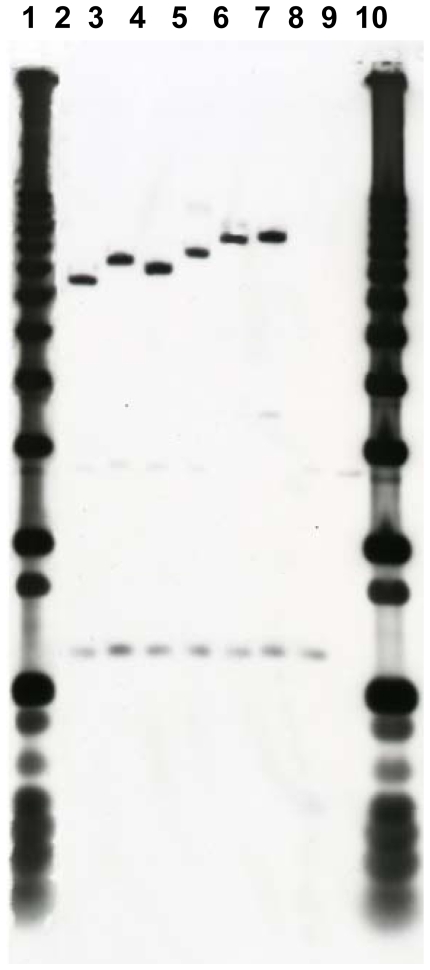
Restriction fragments lenghth polymorphisms (RFLP) obtained after hybridization of DNA ClaI restriction fragments of *S. epidermidis* isolates with a probe for *arcCB* from ACME. Lanes 1) and 10): 1 Kb plus DNA ladder; Lane 2): ClaI-*arcCB* pattern 1 (7+1.2 Kb); 3): ClaI-*arcCB* pattern 2 (7.5+1.2 Kb); 4): ClaI-*arcCB* pattern 3 (7.2+1.2 Kb); 5): ClaI-*arcCB* pattern 4 (8.0+1.2 Kb); 6): ClaI-*arcCB* pattern 5 (8.5+1.2 Kb); 7): ClaI-*arcCB* pattern 6 (9.0+1.2 Kb); 8): ClaI-*arcCB* pattern 7 (1.2 Kb); 9): no hybridization.

For initial characterization of the genetic diversity of ACME found in *S. epidermidis* isolates, chromosomal DNA were digested with a frequent-cutting restriction enzyme *Cla*I and screened for restriction fragment length polymorphisms near the *arc* gene cluster by hybridization with a probe encompassing the ACME-encoded *arcC* and *arcB* genes. We selected 39 (64%) isolates positive for ACME-encoded *arcA* for this analysis. Since a single restriction site for *Cla*I is observed within *arcCB* fragment (inside *arcC*), the hybridization band patterns obtained typically contained two bands, a constant 1.2 kb band, corresponding to a *Cla*I site downstream of *arcC*, and the other band varying in size between 7 and 9 kb, corresponding to a variable *Cla*I site upstream of this gene (Figure 3). A total of seven different *Cla*I-*arcCB* DNA restriction band patterns were identified among the 39 *S. epidermidis* strains carrying ACME (Table 1). Of these, *Cla*I-*arcCB* pattern-6 was the most common (n = 19), followed by pattern-4 (n = 8), pattern-7 (n = 6), patterns -2 and -5 (n = 2 each) and patterns -1 and -3 (n = 1 each).

To further characterize genetic diversity of ACME among the *S. epidermidis* isolates, we used a PCR-based scanning method for amplification of 30 overlapping segments of 1–2 kb in length spanning to the entire archetypal ACME found in USA300; this method allows for a comprehensive assessment of gene content, gene synteny and other structural features of ACME [5]. Among the 65 isolates containing either ACME-encoded *arcA* or *opp-3* genes, 39 distinct PCR scan patterns or variants were identified (Table 1). Of these, 66% (43/65) were classified as ACME-I allotype because they contained both *arc* and *opp-3* gene clusters. Only a single subtype of ACME-I, designated ACME-I variant 02 (abbreviated ACME-I.02), was found in 42% (27/65) of the isolates; the remaining ACME-I subtypes (i.e. subtypes ACME-I.03 to ACME-I.18) were represented by one isolate each. Additionally, there were 18 distinct PCR scan patterns represented by one isolate each that were classified as ACME-II (containing *arc* but not *opp-3* gene cluster) and 4 patterns that were classified as ACME-III (containing *opp-3* but not *arc* gene cluster).

ACME-I.02 was found in *S. epidermidis* isolates recovered from diverse locations, including Argentina, Denmark, Iceland, Hungary, Portugal, Poland and United States. Sequencing of the amplicons that resulted from the PCR scan of five ACME-I.02-positive *S. epidermidis* isolates from different countries yielded a 24,605-bp contig encompassing both the *arc* and *opp-3* gene clusters (Table 1). This ACME-I.02 contig from diverse *S. epidermidis* differed from the archetypal ACME type I variant 01 (abbreviated ACME-I.01) found in USA300 in only 11 nucleotides that corresponded to the open reading frames *SAUSA300_0048* to *SAUSA300_0077* (GenBank accession number NC007793). From the eleven variant sites found, 10 were single nucleotide polymorphisms (6 non-synonymous mutations, 3 synonymous mutations, 1 mutation in non-coding region), and one site involved an inframe insertion/deletion of a 6-bp within a transposase-encoding sequence (*SAUSA300_0060*). Altogether, these results showed that the prevalent ACME-I.02 type in *S. epidermidis* is nearly identical to the ACME-I.01 found in USA300, indicating a recent common origin.

### Estimated Frequency of Horizontal Acquisition of ACME-I.02 in the CC2 Lineage

**Figure 4:**
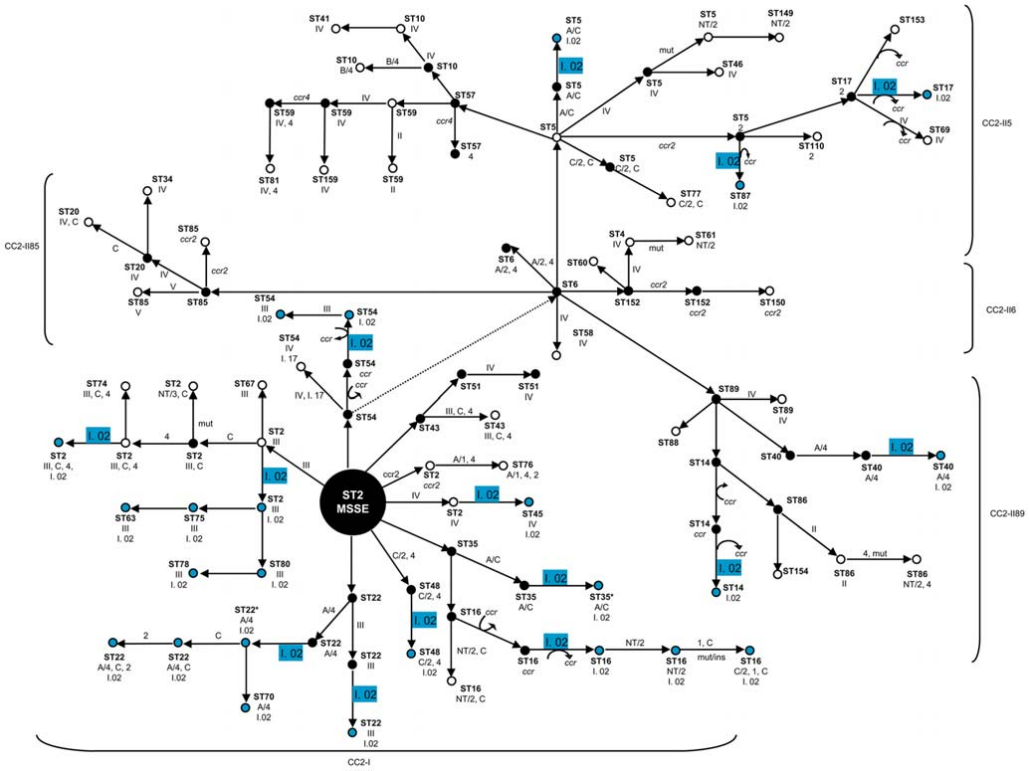
Proposed evolutionary model for ACME acquisition within CC2 clusters and sub-clusters (CC2-I, CC2-II6, CC2-II5, CC2-II85, CC2-II89). Each dot represents a strain with specific characteristics with respect to ST and content of the different mobile genetic elements, namely, SCC*mec* (represented by types I-VI), SCC non-*mec* (represented by the allotype of *ccr*) and ACME types (represented by Roman numbers followed by Arabic numbers). The occurrence of genetic events involving a single MLST locus variation and/or SCC and ACME acquisition/deletion are indicated by arrows and the elements involved in the event are shown next to the arrow. Blue and white dots represent strains found within the collection studied, and black dots represent hypothetical *S. epidermidis* strains. ACME I.02 acquisition is represented in blue.

All 27 *S. epidermidis* isolates containing ACME-I.02 belonged to the prevalent CC2 lineage (Table 1). ACME-I.02 was distributed unevenly between the two clusters that comprised CC2: cluster I of CC2 (abbreviated CC2-I) contains 21 (78%) isolates, and cluster II of CC2 (CC2-II) contains 6 (22%) isolates. To estimate the number of independent horizontal acquisitions of ACME-I.02 within CC2, an evolutionary model was constructed based on the genetic relationships revealed by the eBURST when applied to MLST data (see Figure 4 and Table 1). According to the model proposed, ACME-I.02 was estimated to have been acquired at least on 15 different occasions by strains belonging to CC2 lineage, suggesting frequent mobility of ACME-I.02 within but not beyond this *S. epidermidis* lineage.

## Discussion

In the present study we found that 52% (65/127) of *S. epidermidis* isolates representing the broad genetic and geographic diversity of the species contained one of three ACME allotypes. There were extensive genetic diversity found in ACME islands of *S. epidermidis*, with 39 distinct variants identified by a PCR-based scanning method. Only one of these variants was represented by more than one isolate in the *S. epidermidis* population; this variant, ACME-I.02, contained both the *arc* and *opp-3* gene clusters. All the other variants of ACME are likely to derive from the ancestral ACME-I.02 variant. ACME-I.02 was found in 21% (27/127) of the isolates recovered from seven countries. Importantly, a 24-kb DNA fragment of ACME-I.02 in five *S. epidermidis* isolates was virtually identical to a homologous contig of the ACME-I.01 variant found in USA300, suggesting the interspecies transfer of ACME from *S. epidermidis* into USA300. A similar observation was made for the interspecies transfer of SCC*mec* type IV from *S. epidermidis* strains to *S. aureus*, indicating that *S. epidermidis* provides a reservoir for genetic exchange with *S. aureus* [3].

The observation that the nearly identical ACME-I.01 and ACME-I.02 variants are prevalent among the most widely disseminated lineages of *S. aureus* (i.e. USA300) and *S. epidermidis* (i.e. CC2) suggests that these specific ACME allotypes may confer a particularly high biological fitness advantage. Several lines of evidence suggest that this fitness advantage is not associated to a higher capacity of causing disease. The high prevalence of ACME among *Staphylococcus* species that are common commensals of the human skin, e.g. *S. epidermidis*, S. *capitis* and *S. haemolyticus* [4], [9], together with the fact that ACME was not found specifically associated with disease-causing isolates when compared to colonizing isolates of *S. epidermidis* and *S. haemolyticus* [9], suggest that this element is unlikely to contribute to the capacity of coagulase-negative staphylococci to cause disease in humans. Moreover, ACME was found not to contribute to the capacity of USA300 to cause skin abscess and necrotizing pneumonia in rat infection models [8]. An often overlooked feature of bacterial pathogenicity is the capacity to grow and survive within the host, thereby allowing for enhanced transmission. In this regard, ACME was shown to contribute to the growth and survival of USA300 in the rabbit and in the gastrointestinal tract of the mouse [5], [10]. Furthermore, USA300 was found to be frequently recovered from axilla, inguinal, perineum and rectum [17], [18], which are not common sites of colonization for *S. aureus*. These body sites are usually colonized ubiquitously by *S. epidermidis*, *S. capitis* and *S. haemolyticus*, which exhibit a high frequency of ACME carriage [19], [20]. The acquisition of ACME by *S. aureus* might have allowed for the expansion of its typical colonization niches, providing new opportunities for transmission and dissemination. Altogether, these findings point to a potential role of ACME in conferring a fitness advantage for colonization and transmission rather than an enhanced capacity for infection.

These data also provide evidence for extensive intraspecies transfer of ACME, SCC*mec*, and other SCC elements among *S. epidermidis*, perhaps owing to the fact that 81% of the *S. epidermidis* population carry *ccr* gene complexes (Table 1 and Figure 1). Particularly, a high rate of intraspecies transfer of ACME-I.02 variant within the CC2 lineage was observed, which could be explained not only by the multiple *ccr* gene complexes frequently carried by these strains, but also to an enhanced capacity to accommodate multiple mobile elements, including ACME, SCC*mec* and other SCC elements, within the *orfX* [13]. This together with a high rate of recombination events, previously observed to occur frequently within CC2 lineage [15], may allow for the generation of the extensive genetic diversity among ACME islands afforded by strains belonging to this clonal lineage.

Although the role of *S. epidermidis* species as reservoir and donor of virulence and antibiotic resistance determinants to *S. aureus* is becoming unequivocal, the circumstances that favor the transfer of SCC elements between these two species is not completely understood and should be the focus of future studies. The understanding of the mechanisms and physiological conditions in which such transfer occur would provide us with fundamental tools to help to prevent the emergence of epidemic MRSA strains such as USA300.
